# Lifetime number of affective episodes and functioning in a cohort of patients with bipolar disorder: A cross-sectional study

**DOI:** 10.1192/j.eurpsy.2023.831

**Published:** 2023-07-19

**Authors:** L. Colomer, G. Fico, F. Gutiérrez, E. Pujal, N. Baldaquí, A. Murru, E. Vieta

**Affiliations:** 1 Psychiatry and Psychology, Institute of Neuroscience; 2 Bipolar and Depressive Disorders Unit, Institute of Neuroscience, Hospital Clínic de Barcelona, Barcelona, Spain

## Abstract

**Introduction:**

Cognitive impairment has been commonly found in patients with bipolar disorder (BD).^(1)^ Recent evidence supports the view that global and cognitive functioning decrease as a function of number of prior mood episodes, but the relationship is still not clear. ^(2)^

**Objectives:**

We conducted a cross-sectional study to explore the associations between the lifetime number of affective episodes and functioning, in particular, cognitive functioning in a cohort of patients with BD.

**Methods:**

Adult patients with BD were recruited if euthymic for at least 3 months. Socio-demographic and clinical variables were recollected at the baseline evaluation. Functioning was evaluated at baseline with the functioning assessment short test (FAST). The strength of the association between the lifetime number of affective episodes and FAST subscores was explored with Spearman’s correlation test. Linear regression was computed using cognitive functioning as the dependent variable and a set of clinically relevant variables including the lifetime number of affective episodes as independent variables after controlling for illness duration.

**Results:**

261 BD patients were recruited. Patients with a higher number of lifetime affective episodes showed a significant positive correlation with higher FAST global score (r=0.334, p<0.001) and FAST cognitive functioning subscore (r=0.331, p<0.001). At the linear regression, a higher number of affective episodes was associated to worse cognitive functioning (b=0.037, 95%CI [0.011-0.064], p=0.005).

**Conclusions:**

Poor cognitive functioning in BD could be the result of multiple affective relapses. A timely diagnosis with subsequent effective prophylactic treatment may prevent poor functional outcomes in real-world patients with BD.

**Disclosure of Interest:**

None Declared

